# Large Intronic Deletion of the Fragile Site Gene *PRKN* Dramatically Lowers Its Fragility Without Impacting Gene Expression

**DOI:** 10.3389/fgene.2021.695172

**Published:** 2021-07-20

**Authors:** Sebastian H. N. Munk, Vasileios Voutsinos, Vibe H. Oestergaard

**Affiliations:** Department of Biology, University of Copenhagen, Copenhagen, Denmark

**Keywords:** common chromosomal fragile sites, large genes, *PRKN*, parkin, genomic instability, genome editing

## Abstract

Common chromosomal fragile sites (CFSs) are genomic regions prone to form breaks and gaps on metaphase chromosomes during conditions of replication stress. Moreover, CFSs are hotspots for deletions and amplifications in cancer genomes. Fragility at CFSs is caused by transcription of extremely large genes, which contributes to replication problems. These extremely large genes do not encode large proteins, but the extreme sizes of the genes originate from vast introns. Intriguingly, the intron sizes of extremely large genes are conserved between mammals and birds. Here, we have used reverse genetics to address the function and significance of the largest intron in the extremely large gene *PRKN*, which is highly fragile in our model system. Specifically, we have introduced an 80-kilobase deletion in intron 7 of *PRKN*. We find that gene expression of *PRKN* is largely unaffected by this intronic deletion. Strikingly, the intronic deletion, which leads to a 12% reduction of the overall size of the *PRKN* gene body, results in an almost twofold reduction of the *PRKN* fragility. Our results stress that while the large intron clearly contributes to the fragility of *PRKN*, it does not play an important role for *PRKN* expression. Taken together, our findings further add to the mystery concerning conservation of the seemingly non-functional but troublesome large introns in *PRKN*.

## Introduction

CFSs are specific regions of the genome that often fail to replicate before mitosis, which results in chromosome breakage and high mutation rates (Debatisse et al., 2012). Several pan-cancer genome analyses have also revealed that CFSs are hotspots for structural variants in cancer genomes (Beroukhim et al., 2010; Bignell et al., 2010) with large deletions at the center of CFSs and insertions at the borders of CFSs (Li et al., 2020). In addition, DNA double-strand breaks are remarkably recurrent at CFSs in neuronal progenitor cells (Schwer et al., 2016; Wei et al., 2016).

It has become apparent that breakage and mutations at CFSs are due to replication problems caused by transcription of extremely large genes located at CFSs (Le Tallec et al., 2013; Wilson et al., 2015; Pentzold et al., 2018). To understand how transcription of large genes perturb replication, it is important to keep in mind that eukaryotic replication is initiated bidirectionally from origin of replication complexes (ORCs) scattered across the genome. The distance between these complexes thus determines the minimum distance that two opposing replication forks have to travel to complete replication of the region. Most origins of replication are not used during a normal cell cycle, but during replication stress, excess origins of replication are engaged to ensure complete replication of the genome. The process of transcription repositions the ORCs and thereby clears active intragenic regions of replication origins (Gros et al., 2015; Macheret and Halazonetis, 2018). Hence, transcription of extremely large genes clears vast genomic regions of ORCs and in that way suppresses the firing of backup replication origins in these regions, thus impeding genome replication in an indirect manner. Moreover, clashes between transcription and replication machineries may directly challenge replication of CFSs (Helmrich et al., 2011; Oestergaard and Lisby, 2016; Hamperl et al., 2017). Finally, AT-dinucleotide rich regions capable of forming secondary structures can further perturb replication of certain regions within CFSs (Kaushal and Freudenreich, 2019).

One of the most fragile regions of the human genome is called FRA6E. Here, transcription of the 1.4 Mb *PRKN* underlies its fragility (Glover et al., 2017). Intriguingly, the mature *PRKN* mRNA is only 4 kb despite the fact that the RNA polymerase has to synthesize 1.4 Mb of pre-mRNA. This is because *PRKN* as well as other extremely large genes mainly consist of introns (Voutsinos et al., 2018). Despite their unstable nature and scarce coding information, we recently showed that the size of *PRKN* and other extremely large genes at CFSs are conserved in vertebrates, suggesting that the large introns of these genes possess currently unknown biological functions (Pentzold et al., 2018).

The *PRKN* gene product, parkin, is an E3 ubiquitin-protein ligase that plays a key role in removal of damaged mitochondria through mitophagy (Frank et al., 2012). This process prevents excessive production of reactive oxygen species from dysfunctional mitochondria. Inherited mutations in *PRKN* are the most common cause of autosomal recessive juvenile form of Parkinson’s disease, thus emphasizing the neuroprotective importance of *PRKN* (Klein and Westenberger, 2012). Numerous studies suggest that impaired mitophagy is involved in Parkinson’s disease etiology (Frank et al., 2012; Guo, 2012). Additionally, loss or down-regulation of *PRKN* has been associated with various types of cancer (Gupta et al., 2017), and its loss has been shown to result in a switch to aerobic glycolysis, known as the Warburg effect, which is a characteristic of many cancer types (Zhang et al., 2011).

To investigate the functional significance of extremely large introns, we deleted 80 kb of intron 7 in *PRKN* in our model system, the avian cell line DT40. We previously showed that *PRKN* is transcribed and fragile in this cell line (Pentzold et al., 2018). Here, we find that the deletion does not affect *PRKN* expression levels but leads to a drastic reduction in *PRKN* fragility.

## Methods

### Generation of Constructs

All constructs generated in this study are listed in Supplementary Table 1 and all primers plus other DNA oligos used in this study are listed in Supplementary Table 2. The *PRKN* homology arms for C-terminal fluorescent tagging were amplified with the primer pairs VV5/VV6 and VV7/VV8 for the 5′ arm or the 3′ arm, respectively. The Venus-YFP (2YFP) was amplified using VV47 and VV49. All primers were designed to facilitate directional cloning and they were synthesized by TAG Copenhagen. The amplified products were cloned into pCR2.1-TOPO (Invitrogen) and confirmed by Sanger sequencing (performed by Eurofins Genomics).

The fragments for the PRKN 2YFP-tagging construct were then assembled in pBluescript (SK+). Specifically, the 5′ homology arm was inserted as a *Kpn*I *Sal*I fragment, the 3′ homology arm was inserted as a *Bam*HI *Not*I fragment, and a resistance cassette (BSR or PURO) was inserted as a *Bam*HI fragment. Finally, the 2YFP fragment was inserted as a *Xho*I *Sal*I fragment into the *Sal*I site. Correct orientation was confirmed by restriction digest. The resulting constructs were named pVV6 and pVV15 encoding puromycin (PURO) or blasticidin (BSR) resistance, respectively.

To construct the repair template for *PRKN* intron-7 deletion, genomic regions flanking gRNA Target Site 1 (TS1) and gRNA TS2 were first amplified by PCR templated by genomic DNA (gDNA) from DT40 cells to obtain homology arms.

To create the 5′ homology arm extending 2 kb 5′ of gRNA TS1, PCR was conducted on gDNA with the primers 5′ fwd and 5′ rev adapted with *Apa*I and *Bam*HI restriction sites, respectively. Similarly, PCR was conducted on gDNA with primers 3′ fwd and 3′ rev adapted with *Bam*HI and *Xba*I restriction sites, respectively, to create the 3′ homology arm extending 3′ of gRNA TS2. The homology arm fragments were subcloned into TOPO TA vectors (Invitrogen; according to manufacturer’s protocol) and sequenced (Eurofins Genomics).

Then, the 5′ homology arm was subcloned from the TOPO TA vector into pBluescript (SK+) as an *Apa*I-*Bam*HI fragment. Subsequently, the 3′ homology arm was subcloned from the TOPO TA vector into the 5′homology arm-pBluescript as a *Xba*I-*Bam*HI fragment. Finally, the BSR cassette fragment was cloned in as a *Bam*HI fragment. The final construct was sequenced to confirm correct assembly (Eurofins Genomics).

### Cas9/gRNA Constructs

The backbone for the Cas9/gRNA constructs was pX458 (Addgene). The expression of specific target gRNAs was obtained by annealing the oligonucleotides listed in Supplementary Table 2 and integrating them into pX458 at the *Bbs*I site. Correct integration was confirmed by sequencing (Eurofins Genomics). The constructs were named pX458 PRKN TS1 and pX458 PRKN TS2.

### Cell Culture and Transfection

All DT40 cell lines used in this study are listed in Supplementary Table 3. DT40 cells were cultured in RPMI 1640 medium GlutaMAX (Thermo Fisher Scientific) supplemented with 2% chicken serum (Sigma-Aldrich), 8% fetal bovine serum (Thermo Fisher Scientific), 50 U/ml penicillin, 50 μg/ml streptomycin, and 50 μM β-mercaptoethanol at 39°C with 5% CO_2_.

Transfections for targeted integration were performed by electroporation with Gene Pulser Xcell^TM^ (BioRad) with the settings 25 μF and 0.6 kV. Approximately 35 μg of linearized plasmid DNA was used for transfection with the 2YFP targeting construct. For deletion of *PRKN* intron 7, 20 million cells were transfected with 50 μg linearized repair template and 30 μg of each of the two Cas9/gRNA expression vectors (110 μg DNA in total). Transfection with Cas9/gRNA was transient.

For transient expression of the Cre recombinase, 3.5 million cells were transfected with 15 μg plasmid DNA using the nucleofector system developed by Amaxa Biosystems GmbH (Franklin and Sale, 2006).

### Image Cytometry

For quantification of fluorescently tagged protein levels, the Xcyto^®^ 10 image cytometer (ChemoMetec A/S) was used. Cells were stained by Vybrant Ruby Stain (5 μM, V10309, Thermo Fisher Scientific) to exclude dead cells based on their DNA content. Only cells meeting the following criteria were included in the analysis: not in aggregate, circularity > 0.6, and with DNA content of viable cells.

### Reverse Transcription Quantitative PCR (RT-qPCR)

Total RNA was isolated using the GeneJET RNA Purification Kit (Thermo Fisher Scientific). For RNA samples used for analysis of *PACRG* mRNA levels, 1 μg RNA was pre-treated with DNase I (Fermentas) to remove gDNA according to manufacturer’s instructions in the GeneJET RNA Purification Kit (Thermo Fisher Scientific). cDNA was made using RevertAid Premium Reverse Transcriptase (Thermo Fisher Scientific) with random hexamers and oligo(dT) primers.

Each qPCR reaction was performed in triplicates. All the primer pairs used for qPCR are shown in Supplementary Table 2. All the primer pair efficiencies were close to 1 (100%) and within the acceptable range according to the Minimum Information for Publication of Quantitative Real-Time PCR Experiments guidelines (Bustin et al., 2009). qPCR was performed with Maxima SYBR Green/ROX qPCR Master Mix (Thermo Scientific) according to manufacturer’s instructions for three-step RT-qPCR cycling protocol on CFX96 Real-Time PCR Detection System (BioRad). Fold changes were calculated using the 2^–ΔΔCt^ method (Livak and Schmittgen, 2001). In all cases *GAPDH* was used as reference gene.

### Fluorescence *in situ* Hybridization (FISH)

Metaphase spreads were prepared as previously described (Smith et al., 1990; Pentzold et al., 2018). Briefly, cells were treated with DMSO or aphidicolin (APH) (0.3 μM) (Sigma-Aldrich) over 16 h followed by a 3-h treatment of 0.1 μg/ml colcemid (Life Technologies). Next, cells were swelled in 8 ml hypotonic buffer [20% FBS (vol/vol), 15 mM KCl] for 15 min. Subsequently, 1 ml fixation buffer (25% acetone, 75% methanol) was gradually added. Cells were harvested by centrifugation and then resuspended in 10 ml fixation buffer. Cells were stored at −20°C O/N. Finally, the cells were splatted onto the slides to spread the metaphase chromosomes (Pentzold et al., 2018).

FISH was carried out as previously described with minor modifications (El Achkar et al., 2005; Pentzold et al., 2018). Briefly, the probe used for *PRKN* detection was made with the BAC CH261-119N16 from the CHORI library, and the probes for intron 7 detection were made by amplifying ≈10 kb fractions of intron 7 with the primers listed in Supplementary Table 2. Probes were labeled either by biotin or by digoxygenin by using BioPrime DNA Labeling system (Invitrogen). Metaphase spreads had been treated with RNase H (Thermo Fisher Scientific) before they were incubated with probes.

Metaphase spreads were incubated with blocking reagent and Streptavidin-Cy3 (Alexa 594) (1:200), Biotinylated rabbit anti-streptavidin (Rockland) (1:266) for biotin-labeled probe detection and mouse anti-digoxygenin FITC (Interchim) (1:50) and goat anti-mouse (Alexa 488) for digoxygenin-labeled probe detection.

Slides with metaphase spreads were mounted with coverslips using 15 μl of mounting medium containing DAPI (4% n-propyl gallate, 80% glycerol, 1 μg/ml DAPI). Metaphase chromosomes were visualized on a widefield microscope (AxioImager Z1; Carl Zeiss) equipped with a 100× objective lens (Plan Apochromat, NA 1.4; Carl Zeiss), a cooled CCD camera (Orca-ER; Hamamatsu Photonics), differential interference contrast (DIC), and an illumination source (HXP120C; Carl Zeiss).

## Results

### Establishing *PRKN* Intron 7-Deleted Cell Lines

To investigate the role of large introns in genes coinciding with CFSs, we chose to study the *PRKN* gene, which is highly fragile in our model cell line (Pentzold et al., 2018). To enable live-cell detection of parkin protein levels, we first generated a DT40 cell line with *PRKN* endogenously tagged with a Venus-YFP (2YFP) tandem tag in a background where the non-fragile gene *TOPBP1* was endogenously tagged with TFP on one of its three alleles. The resulting cell line thus has the genotype *PRKN*^WT/2YFP^ *TOPBP1*^WT/WT/TFP^ and is referred to as “P2Y-TT”. Following tagging of *PRKN*, the allele remained fragile in response to replication stress and the tagged gene product was expressed at full length (Supplementary Figures 1A–C). This cell line, P2Y-TT, was used as background for all further genetic manipulations unless otherwise stated.

**FIGURE 1:**
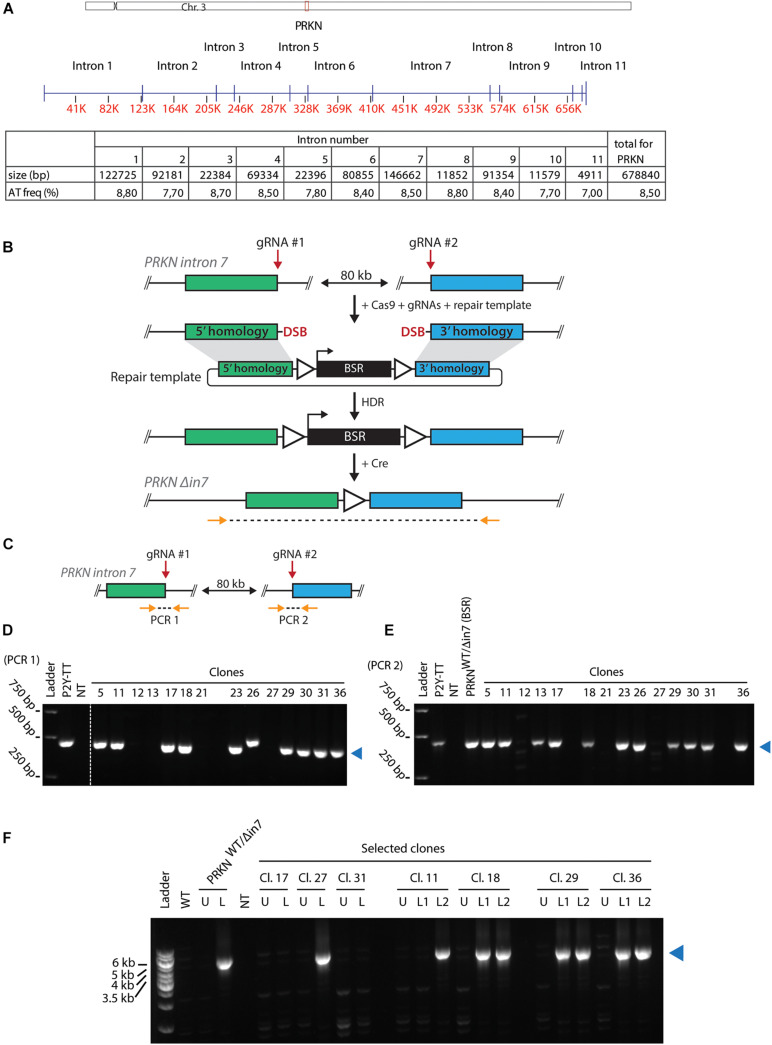
Generating cell lines with large intronic deletion in the fragile site gene *PRKN*. **(A)** *Upper panel*, schematic representation of chicken chromosome 3 drawn to scale. Positions of the centromere (*x)* and *PRKN (red box*) are indicated. *Middle panel*, schematic representation of *PRKN* drawn to scale. Intron numbers are indicated. Exons are represented as vertical pins on a horizontal line. Ruler indicates position along the gene (kb). *Lower panel*, table showing sizes of introns given in basepairs (bp) as well as percentage of AT-dinucleotide frequency (*AT freq*). **(B)** Outline of the strategy for targeted deletion of 80 kb in *PRKN* intron 7. To delete 80 kb in *PRKN* intron 7, cells were transfected with Cas9 and guide RNA (gRNA) expression vectors along with a repair template. The Cas9 nuclease was directed to induce double-strand breaks (*DSB*) at two target sites (*gRNA #1* and *gRNA #2*) 80 kb apart in *PRKN* intron 7. The DSBs can be repaired by homology-directed repair (*HDR*) using the repair template containing a selectable blasticidin resistance (*BSR*) cassette flanked by *loxP* sites (*triangles*) and homology regions (*green and blue rectangles*). After Cre-mediated removal of the floxed BSR cassette, PCR was used to amplify across the 80-kb deletion with primers (*horizontal arrows*) binding at the indicated positions outside of the homology regions. **(C)** Outline of the PCR strategy used to amplify across each of the two gRNA target sites (*gRNA #1* and *gRNA #2*) denoted *PCR 1* and *PCR 2*, respectively. Primers are shown as orange arrows. **(D,E)** PCR amplification across *gRNA #1* **(D)** and *gRNA #2* **(E)**. Analysis of PCR products from the indicated clones and the parental P2Y-TT cell line (positive control). In E, an additional positive control (*PRKN*^WT/Δin7(BSR)^) was included. A no-template control (*NT*) was included in both analyses. A *blue triangle* on the right of each gel indicates the product of the predicted size. **(F)** PCR amplification across the 80-kb deletion (as shown in **B**). Analysis of PCR products from WT cells and selected clones before (*U*) or after (*L*) removal of the BSR cassette. If two clonal populations were tested after loxing, this is indicated with *L1* and *L2. A* positive control with the indicated genotype was also included in the analysis. NT denotes the no-template control. The product of the predicted size is indicated by a blue triangle on the right of the gel.

*PRKN*, which is located on the long arm of gallus chromosome 3, contains 11 introns of varying sizes (Figure 1A). *PRKN* is not enriched for repetitive sequences and replicates in the middle of the S phase (Shang et al., 2013; Pentzold et al., 2018). The total AT-dinucleotide percentage of *PRKN* is 8.5 (Figure 1A, lower panel). We generated cell lines deleted for most of intron 7, which is the largest intron in *PRKN* and has a representative AT-dinucleotide frequency (Figure 1A). Deletion of this intron was achieved by combining a selectable targeting construct, with homology to each side of the region targeted for deletion, with CRISPR/Cas9-mediated cleavage at two target sites flanking the desired 80-kb deletion as outlined in Figure 1B. Successful targeting yields clones with 80 kb of *PRKN* intron 7 replaced with a blasticidin resistance (BSR) cassette. Flanking loxP sites enabled subsequent removal of the BSR cassette. Initial PCR screening suggested that some clones potentially had the 80 kb region replaced by the cassette (Supplementary Figures 2A–E). Subsequently, we tested whether the clones still contained a wild-type *PRKN* allele with PCR analyses of the two guide RNA target sites (Figure 1C). Most clones retained a wild-type allele, but three clones appeared to have lost both wild-type alleles (Figures 1D,E). Then, one potential homozygote and six potential heterozygotes for the 80-kb deletion were transiently transfected with the Cre recombinase to mediate removal of the BSR cassette followed by isolation of single clones. The resulting clones were analyzed with PCR across the region targeted for deletion, and an amplicon of the expected size confirmed successful deletion of 80 kb in *PRKN* intron 7 in a subset of the clones (Figure 1F). The successful homozygote (clone 27) and heterozygotes (clone 11, 18, 29, and 36) are referred to as *PRKN*^Δ^ ^in7/Δ^ ^in7^ and *PRKN*^WT/Δ^ ^in7^, respectively.

Interestingly, in a previous attempt to generate cell lines with the 80-kb deletion, we isolated two clones with the deletion that both turned out to be trisomic for chromosome 3, which is the chromosome that contains the *PRKN* gene (not shown). Thus, we examined the karyotype of the clones derived from this transfection for aneuploidy. Here, we found that 2 out of 14 clones were trisomic for chromosome 3 (Supplementary Figure 2F). This suggests that there is a high risk of chromosome mis-segregation associated with targeting of the *PRKN* gene.

Taken together, the fact that we were able to isolate a homozygous *PRKN* intron 7-deleted DT40 cell line demonstrates that this part of the genome does not contain functional elements essential for cell viability.

### The 80-kb Deletion in *PRKN* Intron 7 Does Not Significantly Change *PRKN* Expression

**FIGURE 2:**
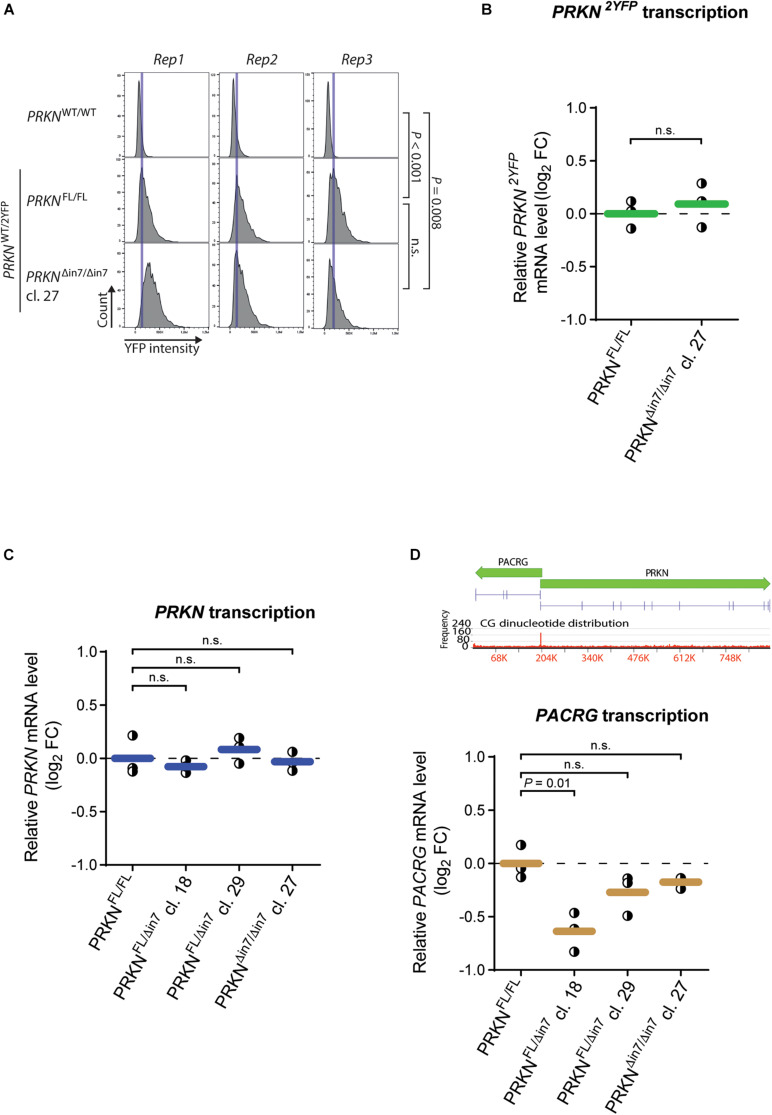
80-kb deletion of intron 7 does not influence *prkn* expression levels. **(A)** Histograms of parkin-2YFP fluorescence levels measured by fluorescence image cytometry in WT cells (*PRKN*^WT/WT^) and the P2Y-TT cell line with full-length *PRKN* (*PRKN*^FL/FL^) or intron 7-deleted *PRKN* (*PRKN*^Δin7/Δin7^*). Rep1-3* denote three individual experiments. Blue vertical lines indicate the histogram peak for P2Y-TT cells in each replicate. Between 900 and 3,800 cells were analyzed per cell line per replicate. *P*-values were calculated using the mean YFP intensities from all replicates with Student’s *t*-test (n.s. = not significant). **(B,C)** RT-qPCR analysis of *PRKN-2YFP* **(B)** and *PRKN* **(C)** mRNA levels in P2Y-TT cells with full-length *PRKN* (*PRKN*^FL/FL^) or intron 7 deletion in one (*PRKN*^FL/Δin7^) or both alleles (*PRKN*^Δin7/Δin7^). Y-axis shows log2 fold change (log_2_ FC) in mRNA levels relative to *PRKN*^FL/FL^. Dots represent individual experiments (*n* = 3), and horizontal lines indicate the mean. Dashed line denotes log_2_ FC = 0. *P*-values were calculated with Student’s *t*-test (n.s. = not significant). **(D)** *Upper panel*, Schematic representation of *PACRG* and *PRKN* including their shared promoter. Gene sizes and orientations are indicated with green arrows. Exons are represented as vertical pins on a horizontal line. Ruler indicates position along the genes (kb). Bar chart showing CG dinucleotide frequency is shown below the genes (2,930-bp windows). *Lower panel*, RT-qPCR analysis of *PACRG* mRNA levels as in **(B,C)**.

Although intron 7 is not essential for cell viability, it may contain regulatory elements that influence *PRKN* expression. We thus asked if deletion of the intron has an effect on parkin levels in the cell. First, we used fluorescence image cytometry to evaluate how *PRKN-2YFP* expression was affected by biallelic intron 7 deletion (Figure 2A). The levels of parkin-2YFP were similar in the intron 7-deleted clone and the parental cell line. Because cells with this genotype must contain the deletion in the 2YFP-tagged *PRKN* allele this indicates that the 80-kb deletion in *PRKN* intron 7 does not alter *PRKN* expression.

To evaluate the effect of intron 7 deletion on *PRKN* transcript levels, we performed reverse transcription quantitative PCR (RT-qPCR) with two different primer sets: One set binding specifically to transcripts from the 2YFP-tagged allele (*PRKN*^2YFP^) and one set binding to transcripts from both the 2YFP-tagged and untagged (*PRKN*^WT^) allele (referred to as “*total PRKN transcripts*”) (Supplementary Figure 3). Only the clone with homozygous intron 7 deletion was included in the analysis of *PRKN*^2YFP^ transcript levels while the homozygote and two heterozygotes were included in the analysis of total *PRKN* transcript levels. No change in either *PRKN*^2YFP^ transcript levels (Figure 2B) or total *PRKN* transcript levels (Figure 2C) were detected in any of the clones, supporting that the intron 7 deletion does not alter *PRKN* expression.

We further investigated whether the intron 7 deletion induced changes in the promoter activity of *PRKN*. Specifically, we exploited that *PRKN* shares its promoter (marked by high GC content in Figure 2D, upper panel) with the gene *PACRG* (parkin coregulated) (West et al., 2003), which is transcribed in the opposite direction of *PRKN*. Thus, we would expect changes in the promoter activity to affect both genes, and we therefore extended our RT-qPCR investigations to include *PACRG* transcription (Figure 2D). While a significant decrease in *PACRG* transcript levels were detected in *PRKN*^*WT*/Δ^ ^in 7^ clone 18 compared to the parental cell line, no significant differences were detected in any of the other clones including the homozygote for intron 7 deletion, suggesting that the difference seen in one of the clones is due to clonal variation.

Altogether, this indicates that the 80-kb deletion in *PRKN* intron 7 does not markedly alter *PRKN* or *PACRG* expression.

### Truncation of *PRKN* Significantly Reduces Its Fragility

**FIGURE 3:**
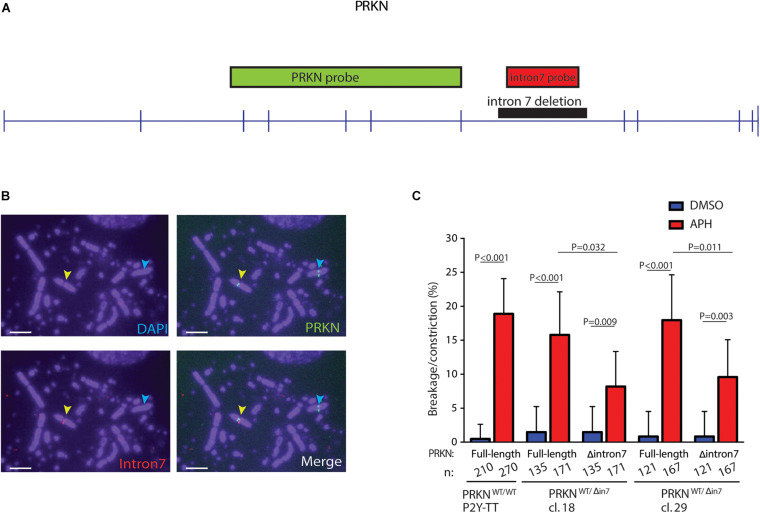
80 kb deletion in intron 7 significantly affects *prkn* fragility. **(A)** Scaled schematic representation of *PRKN*. Positions of the FISH probes for *PRKN* are indicated with *green* and *red boxes*. The deleted region of intron 7 is indicated by a *black box*. Exons are represented as *vertical pins* on a *horizontal line*. **(B)** Representative images of metaphase spreads with FISH probes against *PRKN* (*green*) and *PRKN* intron 7 (*red*) on DAPI-stained metaphase spreads. Cells were treated with 0.3 μM of APH for 16 h before harvesting. *Yellow arrows* point to a break/constriction at the full-length *PRKN* allele and the *cyan arrows* point to the intron 7-deleted *PRKN* allele only recognized by the *PRKN* FISH probe. Scale bars are 5 μm. **(C)** Quantification of breakage/constriction at *PRKN* in P2Y-TT cells and two independently derived clones of *PRKN*^WT/Δ^ ^in7^. Cells were treated with DMSO or 0.3 μM APH for 16 h. n denotes the total number of the indicated *PRKN* allele that was quantified for each clone. The data were analyzed by Fisher’s exact test. Error bars indicate 95% confidence intervals.

To test the hypothesis that the large introns in *PRKN* are underlying its fragility, we performed FISH on metaphase spreads from the two *PRKN*^WT/Δ^ ^in 7^ clones after inducing replication stress by treatment with the DNA polymerase inhibitor aphidicolin (APH). These clones enabled us to use the full-length wild-type allele of *PRKN* as an internal control. For FISH, we used a probe that binds *PRKN* outside of intron 7, which detects both full-length and intron 7-deleted *PRKN*, as well as a probe that binds the deleted region of intron 7 and therefore only detects the full-length *PRKN* (Figures 3A,B). While the full-length *PRKN* locus in the *PRKN*^WT/Δ^ ^in 7^ clones was as fragile as the full-length *PRKN* in the parental cell line, the intron 7-deleted *PRKN* allele was significantly less fragile in both *PRKN*^WT/Δ^ ^in 7^ clones (Figure 3C). Notably, the deletion, which is equivalent to approximately 12% of the length of *PRKN*, resulted in an approximately 50% reduction of the fragility of the gene. Thus, the 80 kb region in intron 7 clearly contributes to *PRKN* fragility even though this region does not have a clear role in *PRKN* expression or cell viability.

## Discussion

Extremely large genes arose in an early vertebrate ancestor due to intron expansions (Voutsinos et al., 2018). Furthermore, the size of extremely large genes seems to be conserved during evolution even though they pose a threat to genome integrity (Pentzold et al., 2018; Voutsinos et al., 2018). In this paper, we have investigated the cellular role of the largest intron in the *PRKN* gene, which is located in a highly fragile CFS (Wilson et al., 2015; Okamoto et al., 2018; Pentzold et al., 2018; Voutsinos et al., 2018). This is to our knowledge the first controlled experiment addressing the function of an extremely large intron. We were able to generate a cell line homozygous for an 80 kb deletion in *PRKN* intron 7, clearly demonstrating that the deleted region is not essential for cell viability. Moreover, we find that this intron 7 truncation does not have any significant effect on *PRKN* gene expression. Yet, the 80-kb intronic deletion leads to an almost 50% reduction of *PRKN* fragility although only shortening the gene length by 12%, which does not appear to be a consequence of altered transcriptional activity. Thus, these 80 kb of intronic sequence with no apparent function are significantly contributing to *PRKN* fragility, most likely reflecting that extreme gene size is a trigger for chromosomal fragility, which suggests a disproportional significance of gene length on chromosomal fragility. The reason for the reduction of fragility upon intron deletion might be that conflicts between transcription and replication are avoided due to the shorter traveling time for the RNA polymerase. However, given the importance of replication timing for fragility, the reduced fragility may result from change in replication timing, which we expect to occur because the transcription unit is shortened and thereby the distance between replication origins at each side of the gene will be reduced. However, further studies are needed to experimentally determine the effect of intron deletion on replication timing. We note that the size of the gene or elements within the intron may play a functional role in certain tissues. It may even be possible that genomic instability at CFSs play a physiological role for instance in neurons where it might serve to generate genetic diversity (Schwer et al., 2016; Wei et al., 2016; Voutsinos et al., 2018). Alternatively, the replication difficulties induced by long introns may provoke epigenetic diversification as shown for replication problems induced by G4 quadruplex forming DNA sequences (Schiavone et al., 2014).

Here, we find that intronic truncation does not lead to changes in gene expression, thus adding to the mystery regarding the conservation of large introns. Therefore, further studies are needed to unravel the functional significance of large introns in genes at CFSs that clearly cause problems for dividing cells.

## Data Availability Statement

The original contributions presented in the study are included in the article/Supplementary Material, further inquiries can be directed to the corresponding author/s.

## Ethics Statement

Ethical review and approval was not required for the animal study because the DT40 parental bursal lymphoblast cell line is commercially available from ATCC.

## Author Contributions

SM performed the 80-kb deletion of PRKN intron 7 and most of the experiments that relate to this. VV constructed the cell lines expressing tagged parkin and generated the intron 7 FISH probe, and also assisted with supervision and data analysis. VO conceived, coordinated, and supervised the project. VO, SM, and VV wrote the manuscript. All authors contributed to the article and approved the submitted version.

## Conflict of Interest

The authors declare that the research was conducted in the absence of any commercial or financial relationships that could be construed as a potential conflict of interest. The reviewer VB declared a shared affiliation with one of the authors SM to the handling editor at the time of review.
