# Supplementary material for: Large Intronic Deletion of the Fragile Site Gene PRKN Dramatically Lowers Its Fragility Without Impacting Gene Expression
Source: Front Genet. 2021 Jul 20;12:695172. doi: 10.3389/fgene.2021.695172 (PMC8329550; doi:10.3389/fgene.2021.695172)
Supplement: Supplementary file 1 [file Data_Sheet_1.PDF]

Supplementary Figure 1

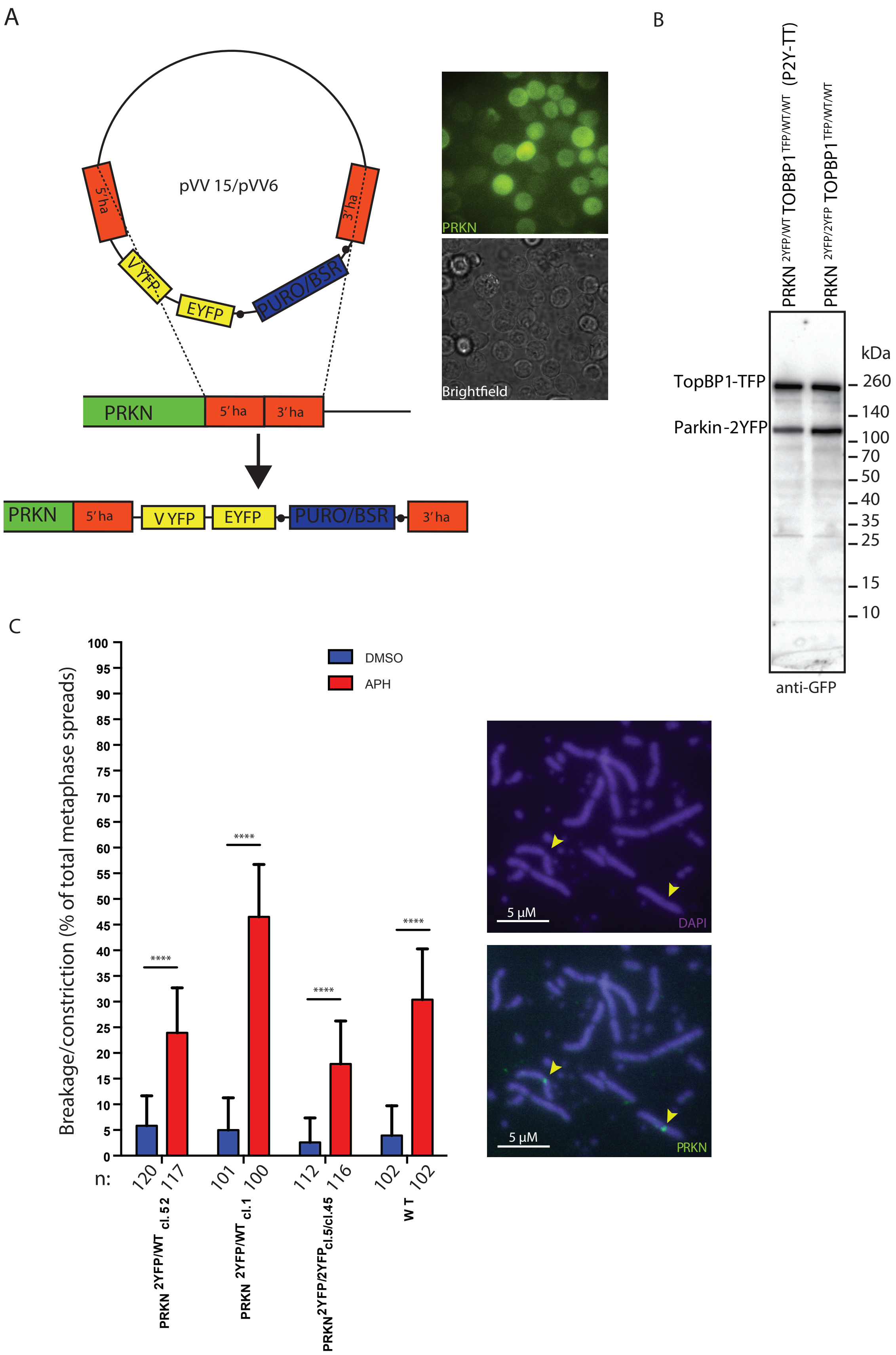

Supplementary figure 1. Endogenous tagging of *PRKN* with Venus-YFP (2YFP) tandem tag
A. *Left*, Schematic representation of the 2YFP-tagging strategy for endogenous *PRKN*. *5'ha*, 5' homology arm; *3'ha,* 3' homology arm. *Thick dots* represent loxP sites. *Right*, representative images of the tagged DT40 cell line. **B.** Western blot with GFP antibody to detect levels of parkin-2YFP in extracts from the indicated DT40 cell lines. TopBP1-TFP is recognized by the same antibody and used as a loading control. **C.** *Left*, Quantification of breaks/constrictions at *PRKN* in the indicated DT40 cell lines. Cells were treated with DMSO or 0.3 µM APH for 16 hours before harvest. The number of metaphase spreads (*n*) that was quantified for each cell line is indicated. The data were analyzed by Fisher's exact test. *Error bars* indicate 95% confidence interval. **** $p < 0.0001$. Right, Representative images of FISH with probe against *PRKN* and DAPI staining on DT40 metaphase spreads. Yellow arrows point to a break at the *PRKN* locus.
