# Supplementary material for: Large Intronic Deletion of the Fragile Site Gene PRKN Dramatically Lowers Its Fragility Without Impacting Gene Expression
Source: Front Genet. 2021 Jul 20;12:695172. doi: 10.3389/fgene.2021.695172 (PMC8329550; doi:10.3389/fgene.2021.695172)
Supplement: Supplementary file 2 [file Data_Sheet_2.PDF]

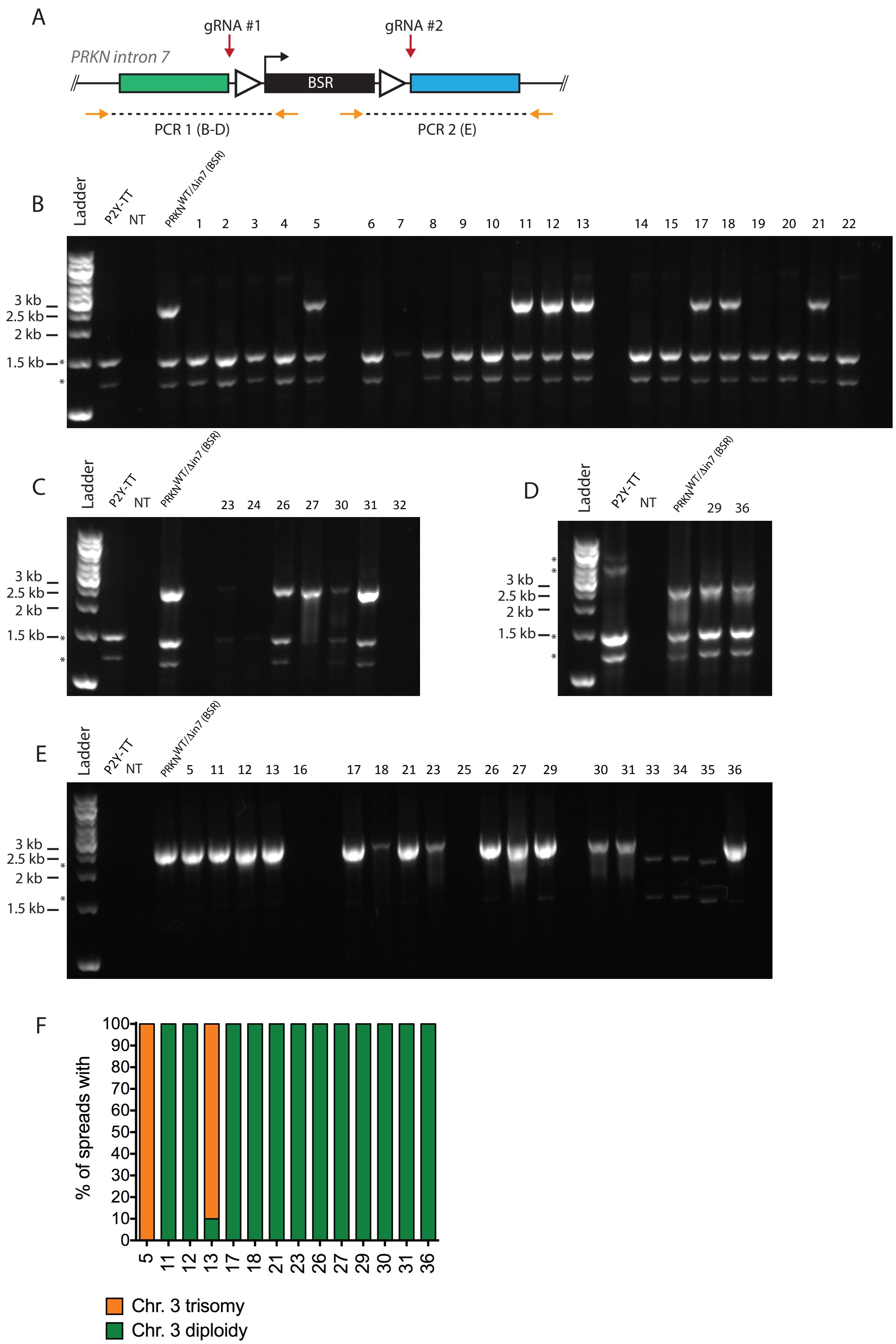

Supplementary figure 2. Validation of *PRKN intro*n 7 deletion

A. Outline of the PCR screening strategy for detecting clones with 80-kb deletion in *PRKN* intron 7. PCR was used to detect correct repair template integration with primers (*orange arrows*) annealing inside the resistance cassette (*BSR*) and outside of the homology regions (*green and blue rectangles*). PCR amplicons are illustrated as dashed lines between primers. **B-D.** PCR detection of repair template targeting at target site 1 *(gRNA #1*). Analyses of PCR products from the indicated clones (*numbers*), P2Y-TT cells, and a positive control (*PRKN*$^{WT/\Delta in7(BSR)}$). *NT* denotes the no-template control. **E.** PCR detection of repair template targeting at target site 2 (*gRNA #2*). Analysis of PCR products from the indicated clones (*numbers*), P2Y-TT cells, and a positive control (*PRKN*$^{WT/\Delta in7(BSR)}$). *NT* is no-template control. For panels B–E, *asterisks* denote unspecific PCR products. **F.** Macrochromosome karyotype analysis of DAPI-stained metaphase spreads from the indicated clones. The y-axis shows the percentage of metaphase spreads from each clone with two (*green*) or three (*orange*) chromosome 3s. 10-15 metaphase spreads were analysed per clone.
