# Supplementary material for: Large Intronic Deletion of the Fragile Site Gene PRKN Dramatically Lowers Its Fragility Without Impacting Gene Expression
Source: Front Genet. 2021 Jul 20;12:695172. doi: 10.3389/fgene.2021.695172 (PMC8329550; doi:10.3389/fgene.2021.695172)
Supplement: Supplementary file 3 [file Data_Sheet_3.PDF]

		mRNA		
		**PRKN**	**PRKN-YFP**	**PARCG**
**primer names**	**Forward**	PARK2_5_F	P2Y_1_F	VV_134
	**Reverse**	PARK2_5_R	P2Y_1_R	VV_135
**amplified part**		Exon 6-Exon 7	Exon 11-Exon 12 with YFP	Exon 1-Exon 2

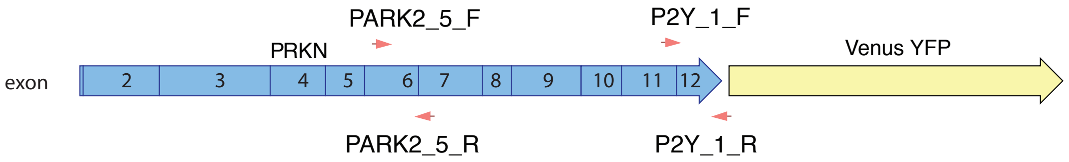

Supplementary figure 3. Primers for RT-qPCR
Upper panel, table showing the names and products of primers pairs used for RT-qPCR. *Lower panel*, schematic representation of PRKN-Venus YFP mRNA with exon numbers and position of primer annealing indicated.
