# Supplementary material for: Large Intronic Deletion of the Fragile Site Gene PRKN Dramatically Lowers Its Fragility Without Impacting Gene Expression
Source: Front Genet. 2021 Jul 20;12:695172. doi: 10.3389/fgene.2021.695172 (PMC8329550; doi:10.3389/fgene.2021.695172)
Supplement: Supplementary file 4 [file Data_Sheet_4.docx]

# Supplementary Material

Supplementary table 1. Constructs used in this study.

| Plasmid | Source |
| --- | --- |
| pVV6 | This study |
| pVV15 | This study |
| Repair template | This study |
| PX458 PRKN TS1 | This study |
| PX458 PRKN TS2 | This study |

# Supplementary table 2. Primers/DNA oligoes

| **Primer** | **Application** | **Sequence (5’-3’)** |
| --- | --- | --- |
| 5’ fwd | Cloning the repair template | GGGCCCAGCCCCAGATTCCCACTTGG |
| 5’ rev | repair template | GGATCCGGCCTCTTGTAATTAGTATGGATTCATT |
| 3’ fwd | repair template | GGATCCGATGTAGCATTTAAGGCTATGTACTGTA |
| 3’ rev | repair template | TCTAGAGGGAGGACAGCCCACAGATT |
| 5'gRNA1 | Guide sequence oligo to TS 1 | CACCGCATACTAATTACAAGAGGCC |
| 5'gRNA2 | Guide sequence oligo to TS 1 | AAACGGCCTCTTGTAATTAGTATGC |
| 3'gRNA1 | Guide sequence oligo to TS 2 | CACCGATGTAGCATTTAAGGCTAT |
| 3'gRNA2 | Guide sequence oligo to TS 2 | AAACATAGCCTTAAATGCTACATC |
| Intron 7_1F | Intron 7 probes | CCCCGGACGAGTCTAGTACA |
| Intron 7_1R | Intron 7 probes | TTGCTGGCTCAGACATCCTG |
| Intron 7_2F | Intron 7 probes | GGATTGCCCTGATAGCCCTC |
| Intron 7_2R | Intron 7 probes | TGCCATAGCTGTTGCACTGA |
| Intron 7_3F | Intron 7 probes | GGGGTTCTTCATCACAGGCA |
| Intron 7_3R | Intron 7 probes | AACGGAGAAAGAGCAGCCTC |
| Intron 7_4F | Intron 7 probes | GTCTACTTGCACGCTGACCT |
| Intron 7_4R | Intron 7 probes | TGATCCACCCCCTCTCCATT |
| Intron 7_6F | Intron 7 probes | AACTATGGGCTGGGGGAGAT |
| Intron 7_6R | Intron 7 probes | GCCAACATGCCAGCAATGAA |
| Intron 7_7F | Intron 7 probes | TCTTCACCCACCATGAGCAC |
| Intron 7_7R | Intron 7 probes | AGTTCCTGGCAGCCATTTCA |
| 5’ of HA5 | Detecting repair template integration/deletion | ACAGGCCTTGCAGATGTTGT |
| BSR rev | Screening for targeted integration | AGAGTGAAGCAGAACGTGGG |
| VO66 | Screening for targeted integration | CACTGCATTCTAGTTGTGGTTTG |
| 3’ of HA3 | Screening for targeted integration | AGCAGAGCAGCCATTGACAT |
| I7_3_F | Screening for targeted integration | TTCTCCCTTCAGCCCTTGGA |
| TS HA5 F | Screening for targeted integration | GTTTTGCCTGTCCATGCGAG |
| TS HA5 R | Screening for targeted integration | AGAAGTATGCAGAGGGAAGGA |
| TS HA3 F | Screening for targeted integration | AGATGTAGGGCTGCCAACTTC |
| TS HA3 R | Screening for targeted integration | GGTGCAGAGAGTTGAACGTG |
| VV_134 | RT-qPCR | GTCTCTGACGGCTTCACTGT |
| VV_135 | RT-qPCR | CGAAATGCTGTGGGCTTTGT |
| P2Y_1_F | RT-qPCR | CCCCAACTGCCATATTCCAG |
| P2Y_1_R | RT-qPCR | CCATCTCGACGTCAAACCAG |
| PARK2_5_F | RT-qPCR | TGGAGCACACCCAACAACTG |
| PARK2_5_R | RT-qPCR | CGATGCATGCACTGGAACAC |
| GAPDH_2_F | RT-qPCR | TCCCTGAGCTGAATGGGAAG |

Supplementary table 3. DT40 cell lines from this study

| Cell line | Source |
| --- | --- |
| PARK2^2YFP/WT^ TOPBP1^TFP/WT/WT^ | This study |
| PARK2^2YFP/2YFP^ TOPBP1^TFP/WT/WT^ | This study |
| PARK2^Δin7_2YFP/+^ TOPBP1^TFP/WT/WT^ | This study |
| PARK2^Δin7_2YFP/Δin7^ TOPBP1^TFP/WT/WT^ | This study |
